# Intracanal bridging ossification after lumbar decompression: a case report and imaging analysis of postoperative bone regrowth

**DOI:** 10.3389/fmed.2026.1948423

**Published:** 2026-09-04

**Authors:** Yudong Fu, Wenhui Zhu, Bifeng Fu, Pengtao Huang, Jinchang Han, Dan Xiao, Wenguang Han, Peng Xu, Jingjing Xue, Xiaoqing Zhang

**Affiliations:** 1 Orthopedics Department, First Teaching Hospital of Tianjin University of Traditional Chinese Medicine, Tianjin, China; 2 National Clinical Research Center for Chinese Medicine, Tianjin, China; 3 Department of Spinal Surgery, Sanmenxia Hospital of Traditional Chinese Medicine, Henan, China

**Keywords:** bone regeneration, bridging ossification, imaging classification, lumbar decompression, osseous restenosis

## Abstract

Postoperative bone regeneration following lumbar decompression is an important factor affecting long-term clinical outcomes. However, bridging ossification between the laminectomy defect and the posterior vertebral body margin has rarely been reported, and its imaging characteristics and underlying mechanisms remain poorly understood. Here, we report a case of bridging ossification following lumbar decompression and discuss its possible mechanisms and imaging manifestations based on the relevant literature. A patient presented in March 2024 with low back pain accompanied by right lower-extremity numbness, pain, and muscle weakness and was diagnosed with lumbar spinal stenosis. The patient underwent right-sided unilateral biportal endoscopic (UBE) decompression at L4–5, after which the symptoms improved and the patient was discharged. In June 2026, the patient developed recurrent radiating pain and numbness in the right lower extremity, with inadequate response to conservative treatment. CT revealed continuous bridging ossification between the original laminectomy defect and the posterior margin of the vertebral body, indicating osseous restenosis. Pulsed radiofrequency neuromodulation was subsequently performed at the right L4–5 foramen, resulting in symptomatic improvement, and the patient was discharged. Based on imaging findings and literature review, this phenomenon may be associated with the combined effects of a pre-existing degenerative osteogenic tendency, postoperative bone remodeling, alterations in local mechanical stress, and degenerative osteophyte formation. This case, together with a review of the relevant literature, summarizes the imaging manifestations of postoperative bone regeneration and may provide a reference for long-term postoperative follow-up, the diagnosis and management of osseous restenosis, and further investigation of imaging patterns of postoperative bone regeneration.

## 1 Introduction

Lumbar spinal stenosis (LSS) is a common degenerative spinal disorder in middle-aged and older adults, and lumbar decompression is a primary treatment for relieving neural compression and improving clinical symptoms. In recent years, minimally invasive techniques such as unilateral biportal endoscopy (UBE) have been increasingly used for lumbar degenerative diseases because of their advantages of reduced tissue injury and faster recovery. However, some patients may experience recurrent symptoms during long-term follow-up, which may be associated with osseous restenosis, scar formation, and segmental degeneration. Among these factors, postoperative bone regrowth is considered an important determinant of long-term outcomes. Previous studies have mainly focused on regrowth from the lamina and bony resection margins, coalescence of bone islands within the decompression defect, and bony overgrowth around the facet joints. In contrast, only limited reports have described bridging ossification within the decompression area, and its clinical manifestations, imaging characteristics, and potential mechanisms remain poorly understood (1). Here, we report a case of bridging ossification between the laminectomy defect and posterior vertebral body margin approximately 2 years after right-sided UBE decompression at L4–5. Based on this case and a review of the relevant literature, we summarize the imaging manifestations of postoperative bone regrowth and explore a potential imaging-based framework for describing postoperative bone regeneration, with the aim of providing a reference for long-term postoperative follow-up, investigation of osseous restenosis, and clinical management.

## 2 Case presentation (FIGURE 1)

A 67-year-old man initially presented in March 2024 with low back pain accompanied by numbness, pain, and muscle weakness in the right lower extremity. Physical examination revealed decreased sensation in the right buttock, lateral thigh, and anterolateral lower leg compared with the contralateral side. The right straight-leg-raising test was positive at 20°, with a positive reinforcement test. Muscle strength was grade IV for right plantar flexion and grade IV for right great-toe dorsiflexion. Based on the clinical and imaging findings, the patient was diagnosed with LSS (Figure 2). Because conservative treatment failed to provide adequate relief, right-sided UBE decompression was performed at L4–5. Adequate neural decompression was achieved intraoperatively (Figure 3), and the patient's symptoms improved significantly after surgery, allowing discharge.

**Figure 1:**
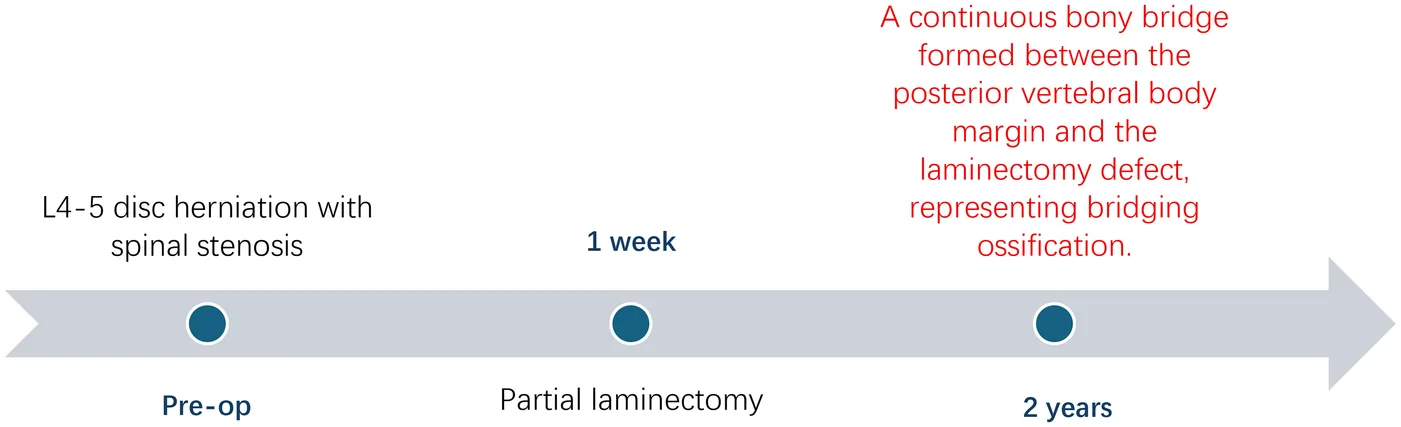
Timeline of the patient's clinical course and key events.

**Figure 2:**
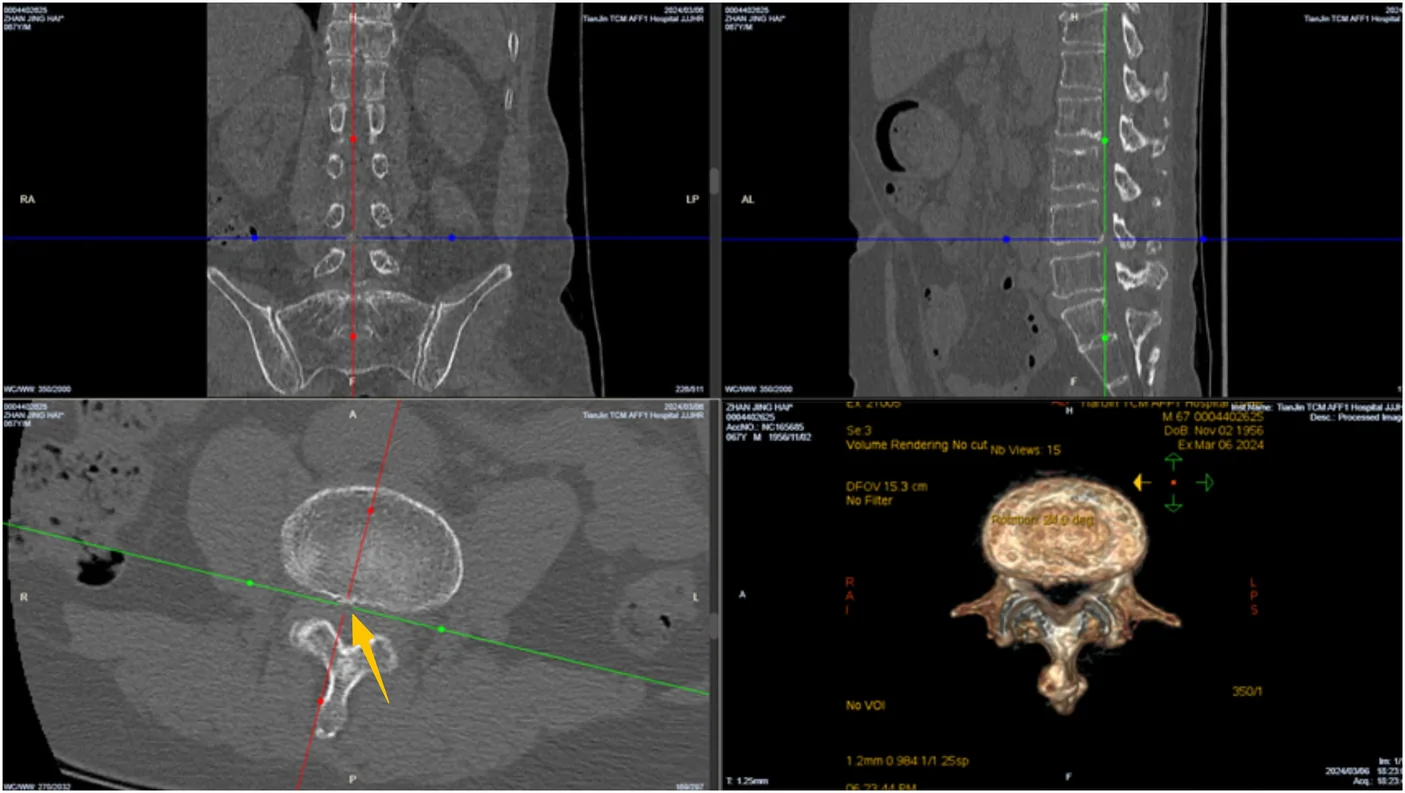
Preoperative imaging findings. The coronal, sagittal, and axial images were positioned at the posterior margin of the inferior endplate of L4, the right margin of the L4 spinous process, and the level of the L4 inferior endplate, respectively; the same positioning criteria were used for the subsequent images. Owing to differences in patient positioning and image slice thickness, the three imaging planes were not perfectly identical. Sagittal imaging demonstrated a large disc herniation at L4–5. Abnormal ossification was observed along the posterior margin of the vertebral body (arrow).

**Figure 3:**
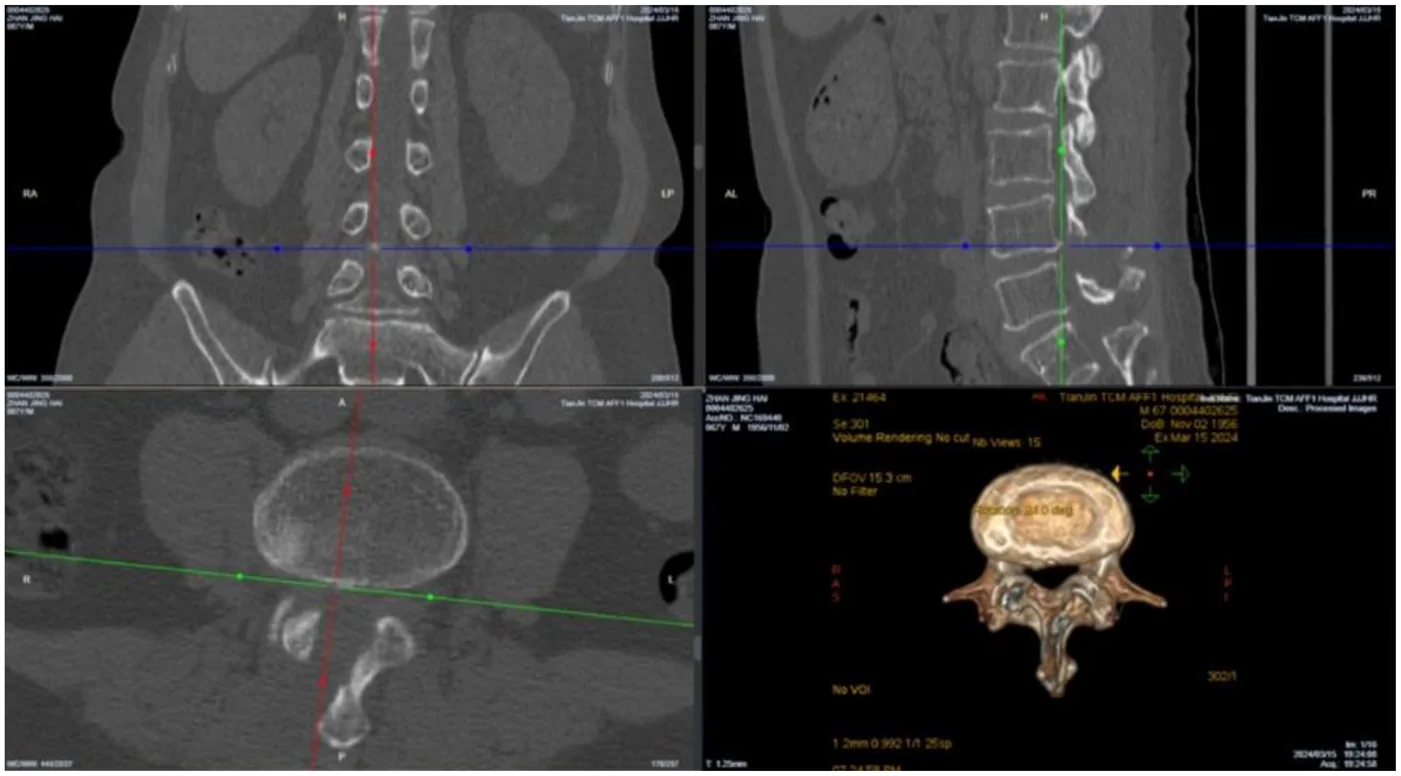
Imaging findings 1 week after surgery. Partial resection of the right L4 lamina was observed, with satisfactory decompression of the spinal canal.

**Figure 4:**
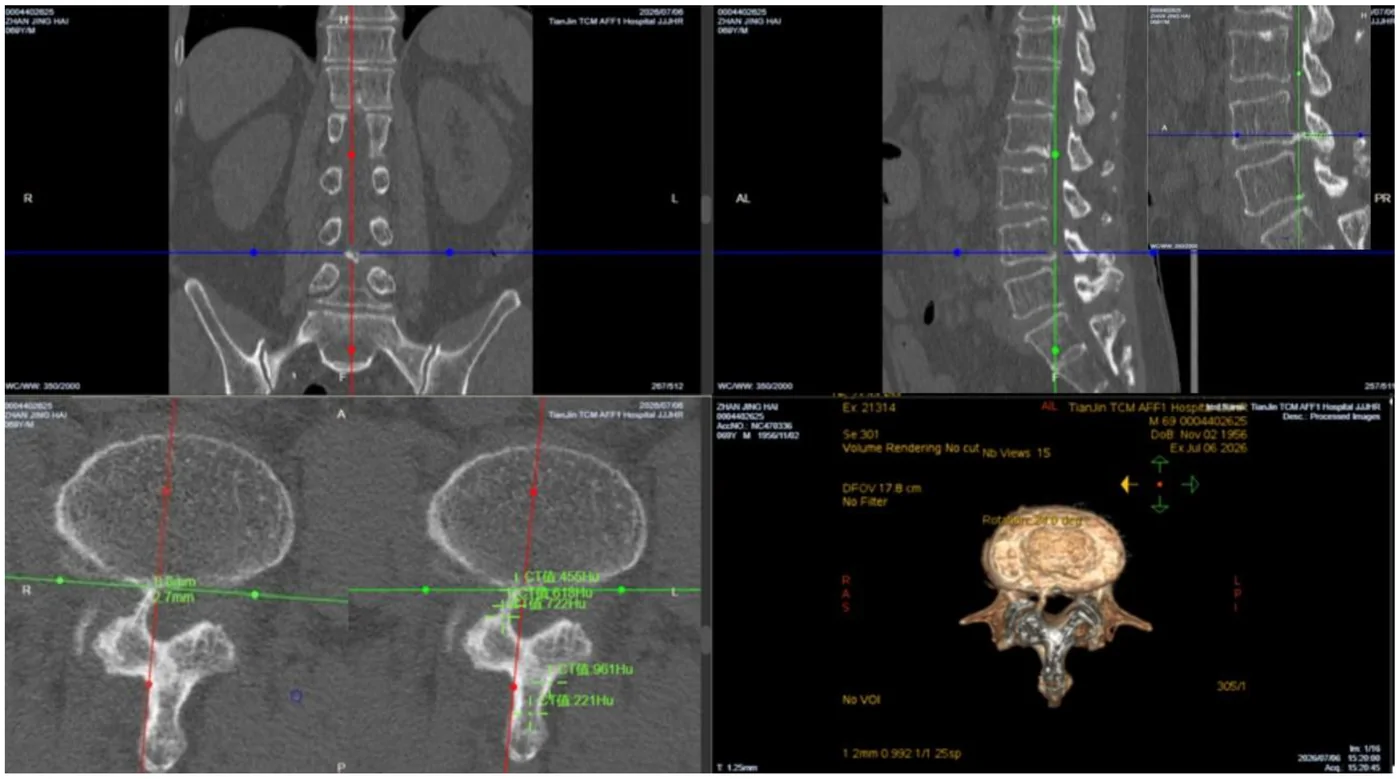
Imaging findings approximately 2 years after surgery. The left axial image shows the measurement of the bridging structure at the corresponding anatomical level, while the right sagittal image shows the slice with the greatest vertical extent. Bone regrowth was observed at the L4 laminectomy defect, forming a continuous bridging bony structure with the posterior margin of the L4 vertebral body. The CT attenuation values were 455 HU, 618 HU, and 722 HU at the ventral, middle, and dorsal portions, respectively, with a mean value of 598 HU. This value was intermediate between the cortical bone (961 HU) and cancellous bone (221 HU) of the contralateral spinous process at the same level, supporting the bony nature of the bridging structure. The structure measured approximately 8.8 mm in the anteroposterior dimension, 2.7 mm in the transverse dimension, and 4.4 mm in height.

Approximately 2 years after surgery, in June 2026, the patient developed recurrent pain and numbness in the right lower extremity. Self-administered nonsteroidal anti-inflammatory drugs and neurotrophic medications provided inadequate relief. Physical examination revealed decreased sensation in the right anterolateral lower leg compared with the contralateral side. The right straight-leg-raising test was positive at 10°, with a positive reinforcement test. Muscle strength remained grade IV for right plantar flexion and grade IV for right great-toe dorsiflexion. Follow-up lumbar CT demonstrated abnormal bony proliferation within the original decompression area (Figure 4), with continuous bridging ossification extending between the laminectomy defect and the posterior margin of the vertebral body, resulting in recurrent spinal canal stenosis. Based on the clinical history and imaging findings, CT-guided pulsed radiofrequency neuromodulation was performed at the right L4–5 foramen, with betamethasone administered at the foraminal region during the procedure. The patient's symptoms improved after treatment, and he was discharged.

## 3 Imaging findings

### 3.1 Discussion

Postoperative bone regrowth after lumbar decompression refers to the formation of new bone within the decompressed area with gradual restoration of bony continuity, which may contribute to delayed osseous restenosis. Postacchini et al. (2) first demonstrated that bone regrowth mainly occurs around the resected lamina and facet joints. In a long-term follow-up study, Chen et al. (3) reported postoperative bone regrowth in approximately 94% of patients, with marked regrowth observed in approximately 15%. Reported patterns mainly include proliferation along the laminectomy margins, facet joint hypertrophy, and new bone formation within the decompression defect. Shimauchi-Ohtaki et al. (4) further reported that marked bony overgrowth may involve the lamina and facet joints and lead to recurrent spinal canal stenosis. In contrast to these previously described patterns, the present case demonstrated new bone extending across the decompression defect and forming a continuous bridging ossification structure between the laminectomy defect and the posterior margin of the vertebral body.

The bridging ossification observed in this case was unlikely to result from a single mechanism but may have reflected the combined effects of a pre-existing degenerative osteogenic tendency, postoperative reparative responses, and local mechanical remodeling. Preoperative CT had already demonstrated a small osteophyte along the posterior margin of the vertebral body, suggesting that, in the context of chronic degeneration and abnormal mechanical stress, the local bone tissue may have exhibited an increased propensity for reactive osteogenesis. During decompression, injury to the lamina, periosteum, and exposed bony surfaces may initiate local inflammatory and reparative responses and activate periosteum-derived osteogenic precursor cells. Meanwhile, osteogenic signaling pathways, including TGF-β/BMP signaling, may promote cellular recruitment, osteogenic differentiation, and bone matrix deposition. Alteration of the posterior spinal elements after decompression may further redistribute local mechanical stress, allowing reparative osteogenesis to undergo continuous remodeling under persistent degenerative and mechanical stimuli. Consequently, newly formed bone may gradually proliferate and extend from different bony margins of the decompression defect, followed by progressive fusion. Through subsequent mineralization and remodeling, the newly formed bone may eventually bridge the original osseous defect and restore an abnormal bony continuity, resulting in the bridging structure observed in this case and contributing to delayed osseous restenosis. Li et al. (1) reported that postoperative bone regrowth may extend beyond the original anatomical boundaries and result in spontaneous osseous fusion, suggesting that postoperative bone remodeling has the potential to form new bony connections, which is consistent with the phenomenon observed in the present case.

Postoperative bone regrowth after lumbar decompression currently lacks a standardized imaging-based classification, and previous studies have primarily evaluated it according to the extent, location, and changes within the decompression defect. Chen et al. (3) graded bone regrowth according to the proportion of the original laminectomy defect that was reossified, whereas Guigui et al. (5) demonstrated by CT that postoperative bone regrowth occurred predominantly within the decompressed area and was particularly evident around the facet joints. Based on previous studies, postoperative bone regrowth can be broadly described as three major morphological patterns: (1) centripetal marginal regrowth, in which new bone gradually extends from the residual lamina, facet joints, or bony resection margins toward the decompression defect; (2) coalescence of bone islands within the defect, in which scattered newly formed bone within the decompressed area progressively enlarges and fuses; and (3) bridging regrowth across the defect, in which newly formed bone crosses the original osseous defect and reconnects separated bony structures. Early studies by Postacchini et al. (2) described regrowth from bony resection margins and new bone formation within decompression defects, while Dohzono et al. (6) further demonstrated quantifiable postoperative bone regrowth around the facet joints. Li et al. (1) reported that newly formed bone could cross the interlaminar space and result in spontaneous osseous fusion, supporting the existence of a bridging growth pattern. In the present case, the newly formed bone extended further across the spinal canal, forming a continuous bony bridge between the posterior decompression area and the posterior vertebral body margin, representing an unusual manifestation of bridging bone regeneration.

Although the patient had developed osseous restenosis, no progressive neurological deficit was identified. Considering the potential contribution of local postoperative inflammatory and scar-related changes to the symptoms, revision decompression was not performed initially. Instead, pulsed radiofrequency neuromodulation combined with local betamethasone injection was performed, with satisfactory symptomatic improvement. Pulsed radiofrequency delivers intermittent radiofrequency electrical fields to modulate neuronal excitability and pain signal transmission while avoiding substantial thermal injury to neural tissue. However, this treatment does not remove the bony stenosis or relieve the underlying mechanical compression. Therefore, the clinical improvement in this case was more likely related to neuromodulation and the local effects of corticosteroid treatment rather than reversal of the osseous stenosis itself. For similar patients without progressive neurological deficits and with pain as the predominant symptom, pulsed radiofrequency combined with local corticosteroid injection may represent a relatively conservative treatment option in selected cases. Nevertheless, its specific role and long-term efficacy in patients with postoperative osseous restenosis require further investigation.

## 4 Limitations

This case has several limitations. First, regular imaging follow-up was unavailable during the approximately 2-year postoperative period, precluding assessment of the onset, progression rate, and specific stages of bridging bone formation. Second, MRI data were unavailable during follow-up, limiting our ability to comprehensively evaluate soft-tissue changes, neural compression, and local inflammatory or scar-related alterations. In addition, this was a single-case observation without histopathological or molecular evidence. Therefore, the proposed involvement of periosteal activation, BMP/TGF-β signaling, and mechanical stress remains largely speculative and is based on the existing literature. Further studies involving larger cohorts and serial imaging are needed to validate these potential mechanisms.

## 5 Conclusion

This case describes a rare manifestation of postoperative bone regrowth following lumbar decompression, in which newly formed bone crossed the original decompression defect and established a continuous bony bridge between the laminectomy defect and the posterior vertebral body margin, resulting in osseous restenosis. Its formation may be associated with a pre-existing degenerative osteogenic tendency, postoperative periosteal injury and reparative osteogenesis, activation of osteogenic signaling pathways such as BMP/TGF-*β*, and redistribution of local mechanical stress, although the underlying mechanisms require further investigation. In patients with recurrent symptoms after lumbar decompression, abnormal bone regrowth and resultant osseous compression should be considered. CT and three-dimensional reconstruction can help delineate the bony morphology and its spatial relationship with the spinal canal. For selected patients with inadequate response to conservative medication but without progressive neurological deficits, pulsed radiofrequency neuromodulation combined with corticosteroid treatment may provide symptomatic relief. Based on the relevant literature, we summarized the major morphological patterns of postoperative bone regrowth and emphasized that, in addition to the extent of regeneration, its growth direction and potential bridging characteristics should also be considered. These findings may provide a reference for the management of recurrent symptoms, long-term postoperative imaging surveillance, and recognition of osseous restenosis.

## Data Availability

The data supporting the findings of this study are available from the corresponding authors upon reasonable request.
